# Molecular Mechanisms of Colistin Resistance in *Klebsiella pneumoniae* Causing Bacteremia from India—A First Report

**DOI:** 10.3389/fmicb.2016.02135

**Published:** 2017-01-09

**Authors:** Agila K. Pragasam, Chaitra Shankar, Balaji Veeraraghavan, Indranil Biswas, Laura E. B. Nabarro, Francis Y. Inbanathan, Biju George, Santhosh Verghese

**Affiliations:** ^1^Department of Clinical Microbiology, Christian Medical CollegeVellore, India; ^2^Department of Microbiology, Molecular Genetics and Immunology, University of Kansas Medical CentreKansas, KS, USA; ^3^Department of Haematology, Christian Medical CollegeVellore, India; ^4^Department of Nephrology, Christian Medical CollegeVellore, India

**Keywords:** colistin, polymyxin B, resistance, *Klebsiella pneumoniae*, whole genome sequencing

## Abstract

Colistin has long been a reserve drug used for the treatment of carbapenem resistant *Klebsiella pneumoniae*. Carbapenem resistance in *K. pneumoniae* has been increasing and is as high as 44% in India. Although a reserve agent, with rise in rates of resistance to carbapenems, the usage of colistin has increased over the years leading to slow emergence of resistance. Colistin resistance is mainly mediated by the alteration in the LPS of bacterial outer membrane with the addition of L-Ara4-N and PEtN molecules. These alterations are mediated by mutations in several genes involved in lipidA modifications and most commonly mutations in *mgrB* gene has been reported. Recently there is emergence of plasmid mediated resistance due to *mcr-1* and *mcr-2* genes which poses a threat for the rapid global spread. This study aims at characterizing eight colistin resistant *K. pneumoniae* from bacteremia by whole genome sequencing. Eight *K. pneumoniae* were isolated from blood culture during 2013 and 2014 at the Department of Clinical Microbiology, Christian Medical College, India. Antimicrobial susceptibility testing was performed and minimum inhibitory concentration (MIC) was determined for colistin and polymyxin B by broth-micro dilution method. Whole genome sequencing was performed using Ion Torrent and the genome of all eight isolates was analyzed. The eight isolates were resistant to all the antimicrobials expect tigecycline. MIC of colistin and polymyxin B were ranged from 4 to 1024 μg/ml and 0.5 to 2048 μg/ml respectively. Multiple mutations were observed in the chromosomal genes involved in lipid A modifications. *mcr-1* and *mcr-2* gene was absent in all the isolates. The most significant were mutations in *mgrB* gene. Among the eight isolates, four, three and one were belonged to sequence types ST 231, ST14 and ST147 respectively. Seven isolates had *bla*_OXA−48 like_, one co-expressed *bla*_NDM−1_ and *bla*_OXA−48 like_ genes leading to carbapenem resistance. Overall, multiple numbers of alterations have been observed. This includes silent mutations, point mutations, insertions and/or deletions. Mutations in *mgrB* gene is responsible for resistance to colistin in this study. Due to emergence of resistance to reserve drugs, there is a need for combination therapies for carbapenem resistant *K. pneumoniae* and colistin must be judiciously used.

## Introduction

*Klebsiella pneumoniae* is a gram negative bacteria and is a common cause of severe community and hospital acquired infections. It readily colonizes human mucosal surfaces especially the gastrointestinal (GI) tract and oropharynx (Bagley, 1985; Dao et al., 2014; Rock et al., 2014). In immunocompromised hosts, it can invade other sites leading to severe infections (Paczosa and Mecsas, 2016). In India, carbapenems are commonly used for treatment of *K. pneumoniae* due to high rates of ESBL producers (Ah et al., 2014). Increasing carbapenem resistance has resulted in colistin (polymyxin E) and tigecycline being used as last resort drugs for treatment of multidrug resistant (MDR) isolates (Carmeli et al., 2010).

Polymyxins are cationic polypeptides with five different compounds, known as polymyxin A–E (Storm et al., 1977). However, only polymyxin B and polymyxin E (colistin) are used in clinical practice to treat bacterial infections. When initially introduced, polymyxins were not widely used due to their adverse effects of nephrotoxicity and neurotoxicity (Falagas and Kasiakou, 2006). However, with the rise of carbapenem resistant organisms, polymyxins have gained attention as one of the few agents available for treatment (Falagas et al., 2005). Polymyxins are positively charged antimicrobial peptide molecules, which act by biding to negatively charged phosphate groups in the lipidA moiety of lipopolysaccharides (LPS). This causes disruption and loss of cell membrane integrity, leading to cell death (Yahav et al., 2012).

Due to its rising and sometimes inappropriate use, colistin resistance is increasing. Resistance to colistin is mainly mediated by modification of the LPS moiety with the addition of positively charged L-Ara4-N and PEtN molecules (Falagas et al., 2010). This modification decreases the negative charge of the outer membrane, reducing its interaction with colistin (Velkov et al., 2013). It is mainly due to mutations in the two component regulatory systems. The most common mutations are in *mgrB, phoP/phoQ, pmrA, pmrB, pmrC*, and *crrABC* (Cheng et al., 2010; Poirel et al., 2015; Wright et al., 2015).

Recently, plasmid mediated colistin resistance named *mcr-1* (plasmid Mediated Colistin Resistance) encoding for phosphoethanolamine transferase has been reported in *Escherichia coli* and *K. pneumoniae* from China (Liu et al., 2016). Following this, *mcr-1.2*, a variant of *mcr-1*, was identified in KPC producing *K. pneumoniae* in Italy (Di Pilato et al., 2016) followed by the *mcr-2* gene reported from Belgium, This had 76% identity with the *mcr-1* gene previously reported (Xavier et al., 2016). The increasing number of resistance mechanisms reported in such a short time period is alarming.

The emergence of colistin resistance is a serious cause for concern for both clinicians and patients, particularly in countries with high rates of carbapenem resistant *Enterobacteriaceae* such as China, India, and Greece. Hence, it is necessary to perform surveillance studies for colistin resistance organisms in Indian hospitals. In this study, we report eight colistin resistant *K. pneumoniae* isolated from bloodstream infections from Christian Medical College, Vellore, India. Whole genome sequencing was employed to study the mechanisms of colistin resistance such as chromosomal genes and plasmid mediated *mcr-1* and *mcr-2* genes.

## Materials and methods

### Bacterial strains

A total of eight clinical isolates of colistin resistant (CR) *K. pneumoniae* (CRKP1–CRKP8) were included in this study. These CR-*K. pneumoniae* were isolated in blood culture from patients admitted at Christian Medical College, Vellore, South India between 2013 and 2015. All the eight isolates were identified up-to species level as per standard biochemical tests (Versalovic et al., 2011) and by Vitek2 (bioMerieux)

### Demographic and clinical details of patients

These were obtained from electronic medical records available in the hospital intranet.

### Antimicrobial susceptibility testing

The Kirby Bauer disk diffusion method was performed to ascertain antimicrobial susceptibility to commonly used agents. This included ceftazidime (30 μg), cefotaxime (30 μg), cefoxitin (30 μg), cefepime (30 μg), piperacillin/tazobactam (100/10 μg), imipenem (10 μg), meropenem (10 μg), ciprofloxacin (5 μg), gentamicin (10 μg), netilmicin (30 μg), amikacin (30 μg), polymyxin B (300 units), and tigecycline (15 μg). The susceptibility results were interpreted as per CLSI (2013, 2014, 2015) guidelines, respectively (CLSI M100S-23; CLSI M100S-24; CLSI M100S-25). Tigecycline results were interpreted according to FDA criteria (http://www.accessdata.fda.gov/drugsatfda_docs/label/2009/021821s016lbl.pdf).

The minimum inhibitory concentration (MICs) were determined by broth micro dilution method (BMD) for both polymyxin agents, colistin and polymyxin B. The results were interpreted as per EUCAST guidelines (EUCAST, 2013, 2014, 2015, respectively), (EUCAST susceptibility breakpoint v 3.0, v 4.0 and v 5.0). *E. coli* ATCC 25922 was used as a quality control.

### Carbapenemase detection by CarbaNP and multiplex PCR

The CarbaNP test was performed for all isolates by the previously described method (Nordmann et al., 2012). *K. pneumoniae* ATCC BAA 1705 and *K. pneumoniae* ATCC BAA 1706 were used as positive and negative controls, respectively.

For molecular testing, all the isolates were grown overnight on blood agar and DNA was extracted using Qiagen kit as per manufacturer's protocol (Qiagen Bacterial DNA mini kit). The presence of carbapenemase genes such as *bla*_SPM_, *bla*_IMP_, *bla*_VIM_, *bla*_NDM_, *bla*_KPC,_ and *bla*_OXA−48 like_ genes were determined by conventional multiplex PCR as described previously (Anandan et al., 2015). The products were analyzed on 2% agarose gel stained with ethidium bromide. Respective positive controls were used for each run (Courtesy: IHMA, Inc., USA).

### Molecular characterization of colistin resistance

#### PCR and sequencing for LPS modifications genes

Genes involved in LPS modification including *mgrB, phoP*/*phoQ*, and *pmrA*/*pmrB*/*pmrD* were amplified and sequenced as previously described (Jayol et al., 2015; Wright et al., 2015).

#### Whole genome sequencing (WGS) for CR- *K. pneumoniae*

WGS was performed using the Ion Torrent PGM platform using 400 bp read chemistry. Sequencing was performed as per the protocol recommended by Life Technologies. Raw reads were assembled *de novo* using Assembler SPAdes software v4.4.0.1 in Torrent suite server version 4.4.3. The genome was annotated using the PATRIC (Pathosystems Resource Integration Center -https://www.patricbrc.org) and RAST (Rapid Annotations using Subsystems Technology) databases. Upon annotation, antimicrobial resistance genes were found as per ARDB and CARD, respectively. These genes were further taken for mutational analysis. The whole genome sequences were deposited at GenBank under the following accession numbers: CRKP1 (*MOXM00000000*); CRKP2 (*MIEJ00000000*); CRKP3 (*MPCT00000000*); CRKP4 (*MDZG00000000*); CRKP5 (*MOXL00000000*); CRKP6 (*MEBR00000000*); CRKP7 (*MOXN00000000*); CRKP8 (*LZYN00000000*).

#### Molecular typing of CR *K. pneumoniae* by MLST

The MLST database at the Center of Genomic Epidemiology was used to identify the sequence types (ST) of the study isolates using WGS data (https://cge.cbs.dtu.dk/services/MLST/).

#### eBURST

The program eBURST v 3.0 was used to identify clonal complexes.

## Results

### Demographic and clinical details of patients

The demographic and clinical details of each patient were obtained from electronic medical records. The patients' ranged from 25 to 59 years in age. Five were male. Six patients were under the care of the hematology team, one was under the care of neurology, and one under nephrology. All but one patient was immunocompromised. *K. pneumoniae* sepsis was hospital acquired in all cases and the sources of infection were neutropenic sepsis *(n* = *4)*, pneumonia *(n* = *1)*, urosepsis *(n* = *1)*, intra-abdominal infection *(n* = *1)*, and line infection *(n* = *1)*. Meropenem was administered to all patients before *K. pneumoniae* was isolated and colistin was administered to five patients. Four patients expired within 30 days of infection and seven within 90 days of infection.

### Antimicrobial susceptibility testing

All isolates were extremely drug resistant (XDR) as per the definition of Magiorakos et al. (2012) All isolates were resistant to cephalosporins, carbapenems, fluoroquinolones, aminoglycosides, and polymyxin B by disk diffusion but were susceptible to tigecycline. The MIC-values determined by BMD for colistin and polymyxin B ranged from 4 to 1024 and 0.5 to 2048 μg/ml, respectively. The MIC results of the eight isolates are summarized in Table 1.

**Table 1:** **Minimum inhibitory concentration (MIC) for colistin and polymyxin B by broth micro dilution (BMD) for ***K. pneumoniae*****.

**MICRO No**.	**MIC (BMD)**	
	**Colistin (μg/ml)**	**Polymyxin B (μg/ml)**
CRKP1	4	0.5
CRKP2	8	32
CRKP3	8	64
CRKP4	16	64
CRKP5	16	64
CRKP6	16	64
CRKP7	16	64
CRKP8	1024	2048

### Carbapenemase detection

CarbaNP test was positive for all the eight isolates tested indicating the production of carbapenemases. Multiplex PCR for carbapenamase genes revealed the presence of *bla*_OXA−48 like_ gene in seven isolates. One isolate co-expressed *bla*_NDM−1 + OXA−232_ genes.

### Whole genome sequence analysis

For whole genome sequencing analysis, PATRIC, and RAST databases have been used to analyse the annotated sequences (http://patricbrc.org/) (http://rast.nmpdr.org/). As these eight isolates were XDR strains, multiple resistance genes against various classes of antimicrobial agents have been identified. These are summarized in Table 2. For cephalosporin resistance, the most common resistance genes were *bla*_TEM_, *bla*_SHV,_ and *bla*_CTX−M−15_. For carbapenem resistance, *bla*_OXA−232_, a variant of *bla*_OXA−48 like_ gene was found in six of the study isolates, one isolate co-producing *bla*_OXA−232_ and *bla*_NDM−1_, while one isolate had *bla*_OXA−181_, also a variant of the *bla*_OXA−48 like_ gene.

**Table 2:** **Antimicrobial resistance genes identified against various antimicrobial agents in CR ***K. pneumoniae*****.

**Isolate**	**β-Lactamase**	**Aminoglycoside**	**Fluoroquinolones**	**Fosfomycin**	**Macrolides**	**Phenicol**	**Rifampicin**	**Sulphonamide**	**Tetracycline**	**Trimethoprim**
CRKP1	TEM-1A, OXA-232^*^, OXA-1, SHV-28, CTX-M-15	*aadA2, aacA4, armA*	*aac(6′)lb-cr, oqxB, oqxA*,	*fosA*	*msr(E), mph(E)*	*catB3*	–	*sul1*	*tet(D)*	*dfrA14, dfrA12, dfrA1*
CRKP2	OXA-1, CTX-M-15, OXA-232^*^, TEM-1B, SHV-28	*aadA2, aacA4, armA*	*aac(6′)lb-cr, oqxB, oqxA*	*fosA*	*msr(E), mph(A), erm(B), mph(E)*	*catB3*	–	*sul1*	*tet(D)*	*dfrA12, dfrA1, dfrA14*
CRKP3	OXA-232^*^	–	–	–	–	–	–	*sul1*	–	–
CRKP4	TEM-1B, CTX-M-15, SHV-12, OXA-232^*^	*aadA2, aacA4*	*aac(6′)lb-cr, QnrS1*	*fosA*	*mph(A), erm(B)*	*catA1*	*ARR-2*	*sul1*	–	*drfA12*
CRKP5	TEM-124, OXA-232^*^, SHV-12, CTX-M-15	*aadA2, aacA4, rmtF*	*aac(6′)lb-cr, oqxA*	*fosA*	*mph(A), erm(B)*	*catA1*	*ARR-2*	*sul1*	–	*drfA12*
CRKP6	OXA-1, CTX-M-15, OXA-232^*^, NDM-1, SHV-28, TEM-1A	*aadA2, aacA4, armA, aph(3′)VIa*	*aac(6′)lb-cr, oqxA, oqxB*	*fosA*	*msr(E), mph(A)*,	*catB3*	*fosA*	*sul1*	*tet(D)*	*dfrA14, dfrA12, dfrA1*
CRKP7	TEM-1B, CTX-M-15, SHV-12, OXA-232^*^	*aadA2, aacA4*	*aac(6′)lb-cr, oqxA, QnrS1*	*fosA*	*mph(A), erm(B)*	*catA1*	*ARR-2*	*sul1*	–	*drfA12*
CRKP8	TEM-1B, CTX-M-15, OXA-181^*^, SHV-11	*strA, rmtF, aacA4*	*QnrB66, oqxB, oqxA, aac(6′)lb-cr*	*fosA*	*mph(A)*	–	*ARR-2*	*sul2*	–	*dfrA12, dfrA14*

* *Variant of bla_OXA−48 like_ gene*.

For colistin resistance, RAST classified 11 genes involved in lipid A modification with L-Ara4N pathway. These included *ugd, arnA_DH, arnA_FT, arnB, arnC, arnT, pmrJ, pmrL, pmrD, pmrM*, and *pmrG*. In addition, 10 genes were classified under genes involved in Lipid A modifications. These include *pagP,pagL, lpxO, phoQ, phoP, pmrA, pmrB, eptA, eptB*, and *yijP*. Of these 21 genes, a few gene sequences could not be retrieved from the annotated genomes of the study isolates from RAST/PATRIC databases and hence analysis was not done. However, for the available genes, sequences were retrieved and analyzed for mutations. Upon analysis, multiple mutations were observed in various genes. In seven isolates, there was a change from arginine to alanine at position 114 of *phoP*. In one isolate, there was a change from arginine to alanine in position 128. In *phoQ*, five isolates had a substitution of glycine for aspartic acid at position 146 and three isolates had the same substitution at position 150. The mutations in other genes such as *pmrB, pmrC, phoB, arnB, arnC, arnT, arnA_FT*, and *pagP* are listed in Table 3 and have not been reported in other studies to date. No mutations were found in *pmrA*, e*ptB*, and *phoR* genes in any of the isolates analyzed. *mcr-1* and *mcr-2* genes were absent in all the study isolates.

**Table 3:** **Cumulative results of amino acid substitutions in various genes contributing to colistin resistance found upon WGS analysis of CR—***K. pneumoniae*****.

**Strain ID**	***mgrB***	***phoP***	***phoQ***	***pmrA***	***pmrB***	***pmrC/ eptA***	***eptB***	***phoB***	***phoR***	***arnB***	***arnC***	***arnT***	***arnA_FT***	***pagP***
CRKP1	None	R 114 A	D 150 G	None	A 246 T L 344 P	V 39 L S 257 L A 279 G	None	None	None	G 47 D A 112 D I 126 V	S 19 T	A 55 G S 56 L A 57 R T 58 Y Y 59 F	I 260 L N 442 K	F 170 I
CRKP2	None	R 114 A	D 146 G	None	None	V 42 L S 260 L	None	None	None	G 47 D A 112 D I 126 V	S 30 T	A 55 G S 56 L A 57 R T 58 Y Y 59 F	I 260 L N 442 K	F 170 T
CRKP3	Premature stop codon (truncated protein 27 amino acid)	R 114 A	D 150 G	None	A 246 T L 344 P	V 39 L S 257 L A 279 G	None	None	None	G 47 D A 112 D I 126 V	S 30 T	A 55 G S 56 L A 57 R T 58 Y Y 59 F	I 260 L N 442 K	F 170 I
CRKP4	Deletion of A at position 10	R 114 A	D 146 G	None	L 344 P	V 39 L R 152 H A 279 G D 477 N	None	None	R 69 C	A 112 D D 285 E	S 30 T	A 55 G S 56 L A 57 R T 58 Y Y 59 F K 372 R I 474 N	L 161 C I 260 L N 442 K	None
CRKP 5	None	R 114 A	D 146 G	None	L 344 P	V 39 L R 152 H A 279 G D 477 N	None	None	R 69 C	A 112 D D 285 E	S 30 T	A 55 G S 56 L A 57 R T 58 Y Y 59 F K 372 R I 474 N	I 260 L N 442 K	None
CRKP6	Premature stop codon (truncated protein 27 amino acid)	R 114 A	D 146 G	None	A 246 T L 344 P	V 42 L S 260 L	None	None	None	G 47 D A 112 D I 126 V	S 19 T	A 55 G S 56 L A 57 R T 58 Y Y 59 F	I 260 L N 442 K	F 170 I
CRKP7	Deletion of A at position 10	R 114 A	D 146 G	None	T 157 P L 344 P	V 42 L R 155 H D 480 N	None	None	R 69 C	A 112 D D 285 E	S 19 T	A 55 G S 56 L A 57 R T 58 Y Y 59 F K 372 R I 474 N	I 260 L N 442 K	None
CRKP8	None	R 128 A	D 150 G	None	R 256 G L 344 P	C 27 F V 39 L V 50 L A 135 P A 279 G	None	None	None	None	S 30 T	A 55 G S 56 L A 57 R T 58 Y Y 59 F L 114 M I 117 V H 156 Q	S 18 A T 185 A I 260 L N 442 K	None

### Targeted gene sequencing analysis of mutations in genes involved in LPS modifications

Of the eight isolates screened for mutations, CRKP3 and CRKP6 had a premature stop codon in *mgrB* gene resulting in a truncated protein of 27 amino acids. Whereas, a frame shift mutation in CRKP4 and deletion of nucleotide A at 10th position of *mgrB* gene in CRKP7 was noted, this could be responsible for the resistant phenotype. Three isolates, CRKP1, CRKP2, and CRKP5 did not have any mutations in the *mgrB* gene. Sequencing of two component systems *phoP/phoQ* and *pmrA/pmrB* and *pmrD* found multiple silent mutations. Since targeted sequencing results did not reveal much information, WGS data analysis was performed to further understand the additional resistance mechanisms.

### Molecular typing of CR *K. pneumoniae* by MLST

MLST database available at https://cge.cbs.dtu.dk/services/MLST/ by the Center of Genomic Epidemiology was used to identify the sequence type (ST) of the study isolates using WGS data. Of the eight CR-*K. pneumoniae*, three different sequence types were identified (ST231, ST14, and ST147). Four isolates belonged to ST231 (CRKP3, CRKP4, CRKP6, and CRKP7), three to ST14 (CRKP1, CRKP2, and CRKP5) and one to ST147 (CRKP8).

### eBURST

**Figure 1:**
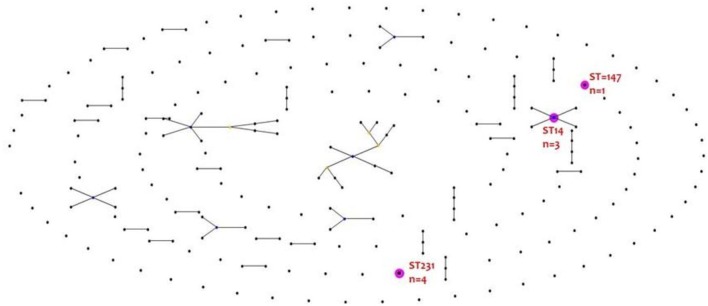
**eBURST analysis of colistin resistant ***K. pneumoniae*****.

eBURST analysis found no clustering of colistin resistant isolates into a clonal complex. The result is depicted in Figure 1.

## Discussion

Antimicrobial resistance is on the rise globally. The limited treatment options available for carbapenem resistant *K. pneumoniae* have resulted in increasing use of colistin, despite its adverse effects. Colistin resistance is now emerging, leaving clinicians with very few options to treat these extensively drug resistant bacteria.

The main challenge in screening colistin resistance lies in determining polymyxin susceptibility (Balaji et al., 2011). Polymyxins are large cationic peptide molecules. They do not diffuse through agar well, hence disk diffusion is neither reliable nor recommended. A reliable screening test is awaited. Other susceptibility methods produce variable and discrepant results. Further, polymyxin B and colistin produce discrepant results (Hindler and Humphries, 2013; Humphries, 2015). Colistin susceptibility testing by disk diffusion has resulted in very major errors of up to 32% when compared with broth or agar dilution methods for colistin (Tan and Ng, 2006; van der Heijden et al., 2007). *E*-test may also produce discrepant results, hence broth microdilution is considered the reference method (Tan et al., 2007).

From this study results, the MIC was found to be two to eight fold higher for polymyxin B when compared to colistin by BMD method. Such discordant results could be due to the non-reliability of MIC testing for polymyxin B. These findings concur with previous studies where the MIC was found to be higher for polymyxin B than colistin (van der Heijden et al., 2007).

A number of studies have reported discrepant results when testing polymyxin B and colistin susceptibility by disk diffusion and MIC methods (Behera et al., 2010). Similar observations were noted in this study; all eight CR-*K. pneumoniae* were found to be resistant on BMD testing with variable MIC values for polymyxin B and colistin as shown in Table 1. Detection of molecular resistance mechanisms are based on preliminary screening with polymyxins. This emphasizes the need for a reliable screening test.

*mgrB* is the commonest chromosomal gene mutation involving modification of LPS which includes insertional inactivation causing colistin resistance. The second commonest cause is multiple mutations in the *pmrAB* and *phoPQ*, the two component systems involved in the LPS modifications.

Of the genes analyzed, mutations in *mgrB* with truncation to 27 amino acids concurs with previous studies (Poirel et al., 2015). The T157P mutation in *pmrB* has also been previously identified (Jayol et al., 2014, 2015; Olaitan et al., 2014). Multiple new mutations were identified in various genes in this study. These include mutations in *phoP* (R114A), *phoQ* (D146G), *pmrB* (L334P, A246T), *pmrC* (V39L, R152H, A279G, D477N, D480N, S260L, C27F, V50L, A235P), *phoR* (R69C), *arnB* (A112D, D285E, G47D, I126V), *arnC* (S30T), *arnA*_*FT* (I260L, N442K, L161C, S18A, T185A), and *pagP* (F170I). However, the significance of these mutations and their contribution to colistin resistance needs to be studied further.

One isolate (CRKP8) had very high MICs of 1024 μg/ml and 2048 μg/ml by BMD for colistin and polymyxin B respectively. This is a huge variation in MIC between two different methodologies.

The clonality distribution of colistin resistant *K. pneumoniae* is of great interest. Worldwide, sequence types (ST) associated with colistin resistant *K. pneumoniae* are ST258, ST512, and ST147 (Mammina et al., 2012; Cannatelli et al., 2014). However, in our study it was ST231, ST14 and ST147. Our study agrees with Jayol et al who reported ST14 *K. pneumoniae* producing *bla*_OXA−48 like_ (Jayol et al., 2014, 2015). However, in this study, the resistance mechanisms were *mgrB* mediated rather than due to mutation in *pmrB*. One of the study isolates belonging to ST14 (CRKP6) was a *bla*_NDM−1_ producer. Isolates with mutated and/or truncated *mgrB* were of different sequence types. This further indicates that resistance determinants are irrespective of the sequence types.

Interestingly, isolates CRKP3 and CRKP6, both of which had a mutation in *mgrB* gene resulting in a truncated product, were not of the same clone. However, isolate CRKP4 and CRKP7 which had a frame shift mutation in *mgrB* gene, both belonged to ST231.

## Conclusion

Inappropriate usage of colistin in the clinical setting should be avoided, as selection pressure may contribute to colistin resistance due to mutations. Further, use of colistin in agricultural practices should be forbidden to prevent the further spread of resistance. The global demand for colistin in agriculture is estimated to reach 16,500 tons by 2021 (QYResearch Medical Research Centre, 2015). India is also one of the leading producers of colistin for veterinary use (Liu et al., 2016). In this study, multiple mutations in the genes coding for LPS modification have been observed. These include silent mutations, point mutations, insertions, and/or deletions. Mutation profiles were observed to be divergent. This signifies the fact that clinical isolates do develop mutations for survival. Altogether, mutations in the *mgrB* gene were found to be the most common, followed by mutations in the other genes such as *pmrB, pmrC, arnB*, and *arnAFT*. This observation emphasizes that colistin resistance is multifactorial. However, the significant effect of these mutations observed in this study needs to be validated. Therefore, further studies should be focused on the role of these genes in pathways involving LPS modifications conferring resistance to colistin.

## Ethics statement

Ethical approval was obtained for the study from the Ethical Committee of Christian Medical College, Vellore, India.

## Author contributions

AP, CS, FI: lab methods, data analysis, manuscript writing. BV, IB: study design and manuscript writing. LN, BG, SV: study design and data collection.

## Funding

Fluid Research Grant of Christian Medical College, Vellore, India.

### Conflict of interest statement

The authors declare that the research was conducted in the absence of any commercial or financial relationships that could be construed as a potential conflict of interest.
